# Supplementary material for: Numerical stability of DeepGOPlus inference
Source: PLoS One. 2024 Jan 29;19(1):e0296725. doi: 10.1371/journal.pone.0296725 (PMC10824456; doi:10.1371/journal.pone.0296725)
Supplement: S1 Appendix — (PDF) [file pone.0296725.s001.pdf]

A Performance at Reduced Precision

The red X signifies the model crashed at these precision values.

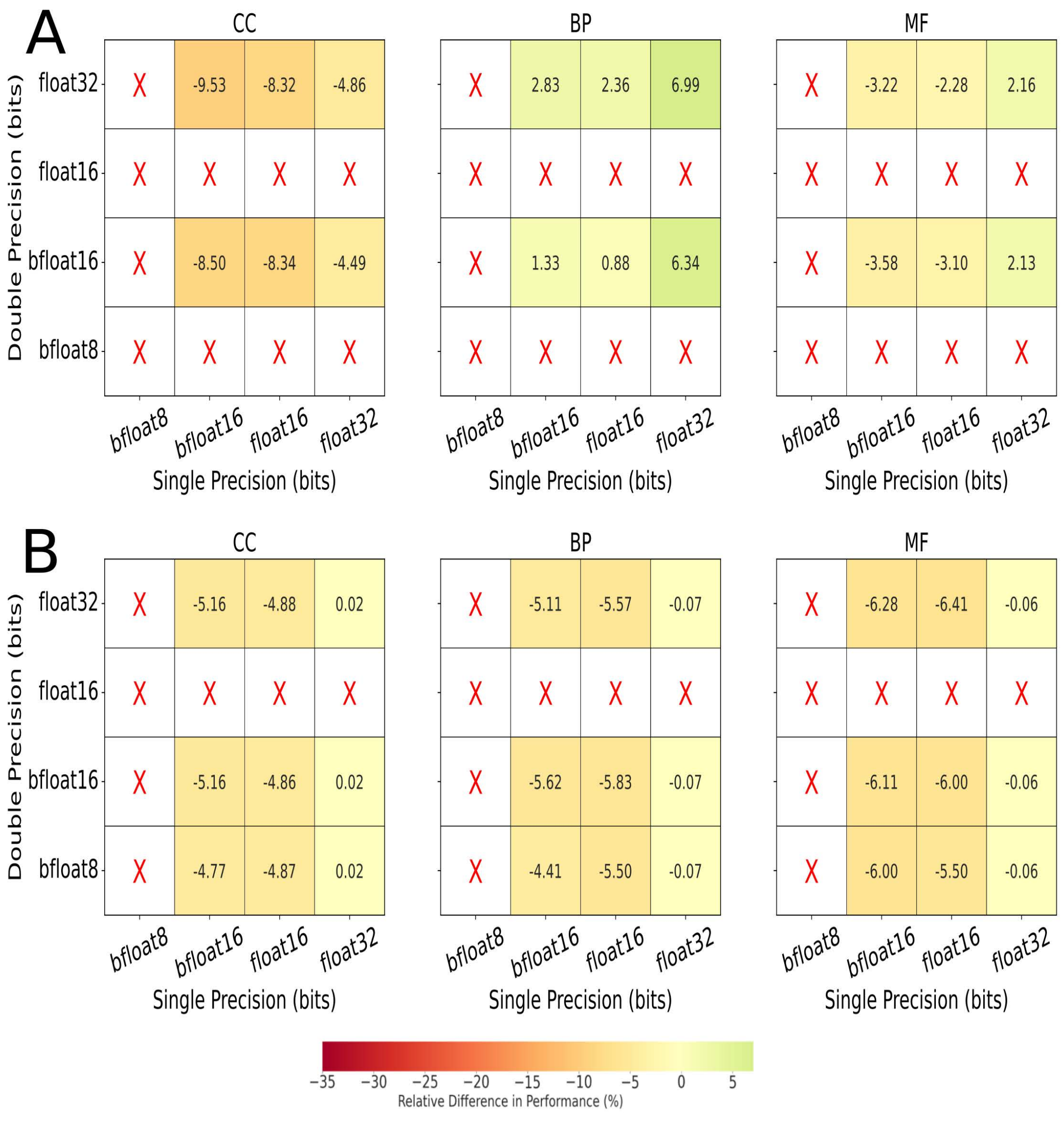

Fig 6. Difference in Fmax Performance between Reduced Precision Formats and IEEE for GO Classes Using Inbound and Outbound Precision Only. Fig. 6 shows at which Single-Precision Value Performance Begins to Drop.

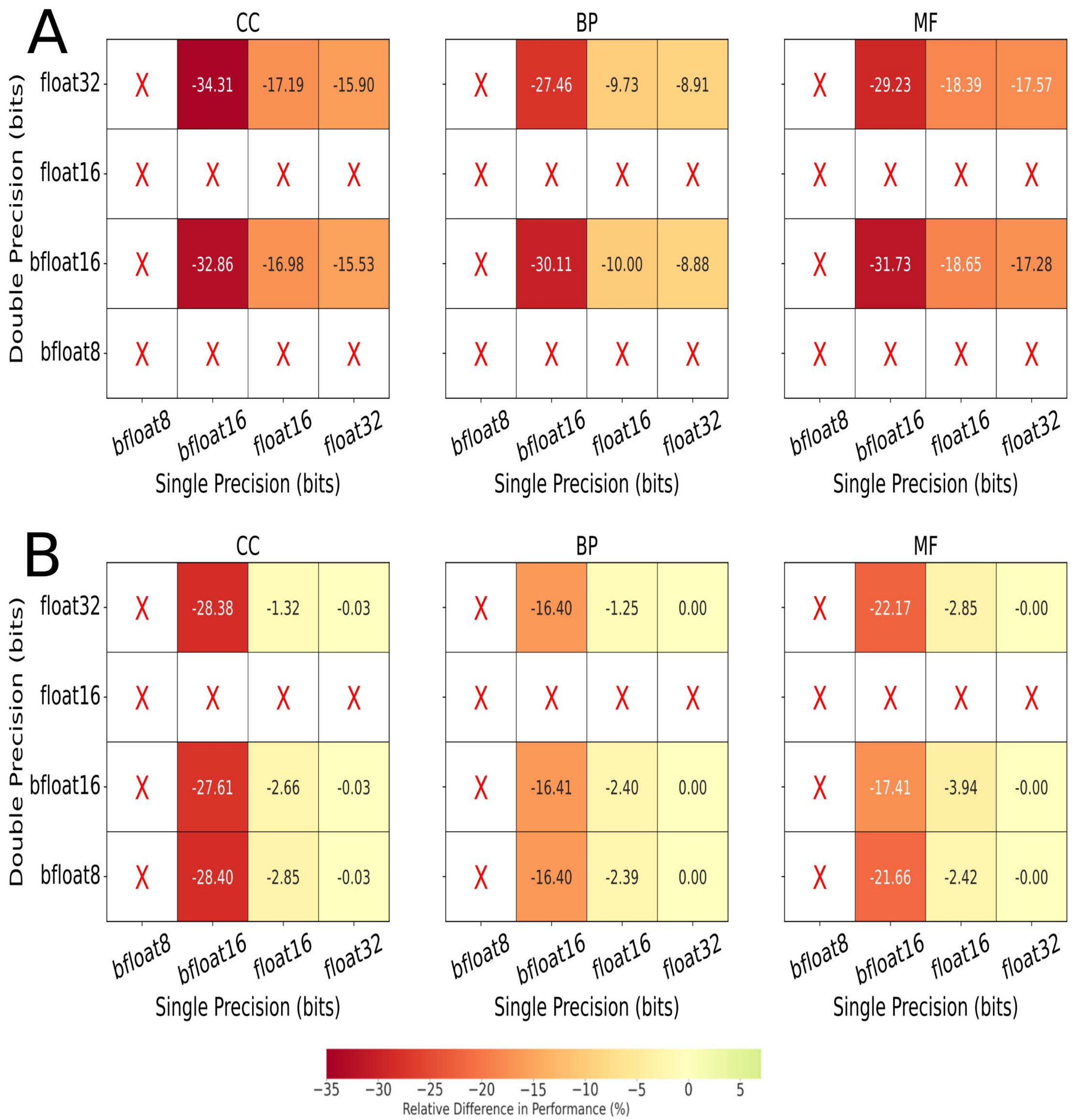

Fig 7. Difference in Smin Performance between Reduced Precision Formats and IEEE for GO Classes Using Inbound and Outbound Precision Only. Fig. 7 shows at which Single-Precision Value Performance Begins to Drop.

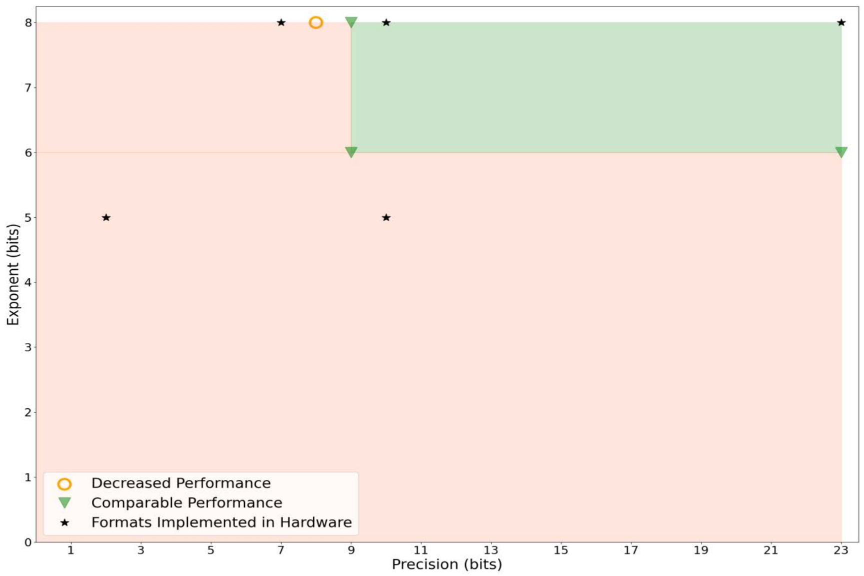

Fig 8. Search for Optimal Precision and Exponent Values Before Performance Drop Off for VPREC Inbound Mode in Single-Precision. Double-precision is reduced to single-precision and the green shaded region marks the area of acceptable values for reduced precision and comparable performance. We observe that drop off in performance for single-precision is not impacted by double-precision being reduced to single-precision.
