# Supplementary material for: Numerical stability of DeepGOPlus inference
Source: PLoS One. 2024 Jan 29;19(1):e0296725. doi: 10.1371/journal.pone.0296725 (PMC10824456; doi:10.1371/journal.pone.0296725)
Supplement: S2 Appendix — (PDF) [file pone.0296725.s002.pdf]

B Performance Metrics With and Without Instrumentation

	Verrou Instrumentation (hh:mm:ss)	IEEE Instrumentation (hh:mm:ss)	Slowdown Factor
Mean	56:50:39	00:00:30	6821.3
Maximum	61:01:05	00:00:32	6864.5
Minimum	55:47:09	00:00:28	7172.5
Standard Deviation	01:32:59	00:00:01	5579.0

Table 5. Comparison of Runtimes Across DeepGOPlus CNN Inference With and Without Instrumentation Obtained Over A Subsample of the Protein Dataset and Averaged Over 10 Iterations.

	Verrou Instrumentation (GB)	IEEE Instrumentation (GB)
Mean	2.67	2.28
Maximum	2.67	2.30
Minimum	2.67	2.27
Standard Deviation	0.00	0.01

Table 6. Comparison of Maximum Resident Set Size Across DeepGOPlus CNN Inference With and Without Instrumentation Reported Over A Subsample of the Protein Dataset and Averaged Over 10 Iterations.
