# Supplementary material for: Numerical stability of DeepGOPlus inference
Source: PLoS One. 2024 Jan 29;19(1):e0296725. doi: 10.1371/journal.pone.0296725 (PMC10824456; doi:10.1371/journal.pone.0296725)
Supplement: Attachment — Submitted filename: Response to Reviewers.pdf [file pone.0296725.s003.pdf]

UNIVERSITÉ Concordia UNIVERSITY

GINA CODY SCHOOL OF ENGINEERING AND COMPUTER SCIENCE

Department of Computer Science and Software Engineering

Montreal,
September 22, 2023

Dear Editor and Reviewers,

We would like to thank you again for considering our manuscript for publication in *PLOS One* and for the thorough review. Please find below the answer to your comments. The corresponding revision of our manuscript is attached, where revisions are highlighted in blue.

We thank you again for your time and consideration, and we remain available for any further information you may need.

Yours sincerely,

Inés Gonzalez Pepe, on behalf of the authors

Signature

Inés Gonzalez Pepe

Comments to the Author

1. Is the manuscript technically sound, and do the data support the conclusions?

The manuscript must describe a technically sound piece of scientific research with data that supports the conclusions. Experiments must have been conducted rigorously, with appropriate controls, replication, and sample sizes. The conclusions must be drawn appropriately based on the data presented.

Reviewer #1: Partly

Reviewer #2: Yes

Reviewer #3: Partly

---

2. Has the statistical analysis been performed appropriately and rigorously?

Reviewer #1: Yes

Reviewer #2: Yes

Reviewer #3: Yes

---

3. Have the authors made all data underlying the findings in their manuscript fully available?

The PLOS Data policy requires authors to make all data underlying the findings described in their manuscript fully available without restriction, with rare exception (please refer to the Data Availability Statement in the manuscript PDF file). The data should be provided as part of the manuscript or its supporting information, or deposited to a public repository. For example, in addition to summary statistics, the data points behind means, medians and variance measures should be available. If there are restrictions on publicly sharing data—e.g. participant privacy or use of data from a third party—those must be specified.

Reviewer #1: Yes

Reviewer #2: Yes

Reviewer #3: Yes

---

4. Is the manuscript presented in an intelligible fashion and written in standard English?

PLOS ONE does not copyedit accepted manuscripts, so the language in submitted articles must be clear, correct, and unambiguous. Any typographical or grammatical errors should be corrected at revision, so please note any specific errors here.

Reviewer #1: No

Reviewer #2: Yes

Reviewer #3: Yes

---

5. Review Comments to the Author

Please use the space provided to explain your answers to the questions above. You may also include additional comments for the author, including concerns about dual publication, research ethics, or publication ethics. (Please upload your review as an attachment if it exceeds 20,000 characters)

Reviewer #1: This paper investigates the opportunity to use reduced-precision floating point formats for DeepGOPlus inference to reduce memory consumption and latency. This is meaningful. However, the current version of the paper is poorly readable.
1. It's recommended to add an overall structure diagram to highlight the innovative points and illustrate the network architecture of the paper.

Answer

As per the recommendation, the above flowchart has been added to the Introduction of the paper.

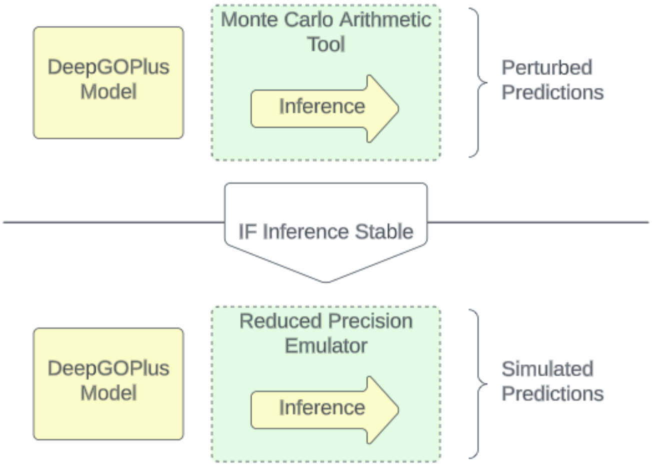

2. The author should improve the format and language (such as formula numbering, etc.) of the paper to increase its readability.

Answer

The paper has been proofread, the mathematical formulas have been numbered and the typos and language has been fixed to improve readability.

Reviewer #2: The article presents a nice approach that demonstrates the numerical stability of a state-of-the-art sequence-based protein function prediction method named ‘DeepGOPlus’. Although the approach is highly interesting there are multiple issues that need to be resolved before the article can be considered for publication in the journal.

Major Issues

1. The approach discussed in the manuscript can be applied to any CNN-based deep learning method for a certain task. The motivation and rationale behind the choice of ‘sequence-based protein function prediction’ as the task is not clear. This obscures the main theme of the article, that in turn confuses the reader, who doesn’t get to understand what is the main message of this article? Please rewrite the ’Abstract’ and ‘Introduction’ sections of the manuscript in a way that this confusion doesn’t arise.

Answer

Clarification as to why sequence-based protein function prediction and the DeepGOPlus model were chosen has been added to the introduction as shown below.

Modified Abstract:

This paper investigates DeepGOPlus, a CNN that predicts protein function. DeepGOPlus has achieved state-of-the-art performance and can successfully take advantage and annotate the abounding protein sequences emerging in proteomics.

Modified Introduction, addition to line 4 and 29:

Advances in the field of proteomics have resulted in an abundance of protein sequences that cannot be identified by traditional experimental means, thus leading to innovations in computational protein function detection methods. Considering a protein sequence is the most common piece of information a researcher can find about a protein, sequence-based protein function prediction has the potential to provide information about little-known or newly discovered proteins, compared to other methods. Moreover, identifying a protein’s function is an important clue to understanding the critical role proteins often play in the human body. Therefore, identifying a protein’s function can lead to increased understanding of the human body’s functions and ailments.

While many proteins are being discovered, little is known about them or their function. Proteins play important roles within organisms as catalysts and drivers of many biochemical reactions, but in order to understand these reactions, the proteins' functions must be known. Understanding the functions of these newly discovered proteins is pivotal, particularly in the context of drug discovery, where manipulating protein behavior through tailored molecules is a core strategy. Leveraging a protein's sequence, which is often the most readily available information, allows for the optimization of drug design.

Automating this process can allow for the large amounts of data being produced to be leveraged to decrease the time needed to design medication and reduce the trial and error of determining how man-made molecules should interact with proteins~\cite{chen2018rise,zhang2017machine}.

Deep neural networks (DNNs) have become popular approaches for protein function prediction in recent years, due to requiring significantly less time and human effort to achieve good performance relative to existing experimental means~\cite{tiwari2014survey,kadam2014prediction,s2013review,le2022potential, kha2022identifying,seo2018deepfam,you2018deeptext2go,you2018golabeler}. Nonetheless, while current DNN models are able to achieve high predictive performance, the numerical reliability of these predictions --- defined as the stability with respect to minor numerical perturbations --- is unknown and should be evaluated for several reasons.

…

This paper centers its investigation on DeepGOPlus~\cite{kulmanov2020deepgoplus} is a state-of-the-art CNN protein function classification model. It is chosen due to being one of the top performers in the CAFA3 challenge~\cite{sapoval2022current}, a community wide challenge to assess computational protein function prediction methods. Additionally, both the model and its data readily available, making it an ideal candidate for study.

~~Focusing on DeepGOPlus~\cite{kulmanov2020deepgoplus}, a state-of-the-art CNN protein function classification model,~~ this This paper aims to (1) determine the stability of DeepGOPlus inference as a result of minute numerical perturbations, (2) evaluate the possibility of using reduced numerical precision formats during inference when using DeepGOPlus.

2. What are some other methods to achieve the two goals discussed in the 3rd paragraph of page 2? What is the reason behind choosing MCA and not these other methods? Please explain.

Answer

Clarification as to alternatives to MCA and why MCA was chosen over these alternatives has been added to the introduction along with motivation as to the use of VPREC.

Modified Introduction, addition to line 34 and 42:

To achieve the first goal, we study the numerical stability of DeepGOPlus by using Monte Carlo Arithmetic (MCA)~\cite{parker1997monte}.

MCA is a stochastic arithmetic technique to empirically evaluate numerical stability by injecting noise into floating point operations involved in programs and quantifying the resulting error at a given virtual precision. Other approaches to determining numerical stability involve formal error analysis and interval arithmetic, but unlike MCA they do not scale to large code bases and require modification of the source code.

We apply MCA to the model through the software tool Verrou~\cite{fevotte2016verrou}, which dynamically replaces floating point operations by perturbed ones at program execution time. The perturbations introduced simulate noise encountered when running code in different environments subject to library updates, OS and/or hardware changes, and allow for assessing the precision of scientific pipelines. To achieve the second goal, we emulate reduced precision formats with the VPREC tool~\cite{chatelain2019automatic} and observe the corresponding predictive performance.

Reviewer #3: Overall, the study presented raises important concerns and provides valuable insights into the numerical stability and precision requirements of DeepGOPlus, a CNN used for predicting protein function. Here are some major concerns on the study:

1. The paper mentions investigating the opportunity to use reduced-precision floating point formats for DeepGOPlus inference, but it does not thoroughly discuss the potential impact of reduced precision on the model's performance and prediction accuracy. The study should have provided a more detailed analysis of how the reduced precision affects the model's output quality and whether any trade-offs exist between precision reduction and prediction reliability.

Answer

In order to provide a more detailed analysis on how reduced precision affects the model’s output, additional experiments were conducted to determine at which precision precisely performance begins to drop and where in the code the numerical instability is originating from . Below is the figure added that demonstrates our findings regarding reduced precision for DeepGOPlus.

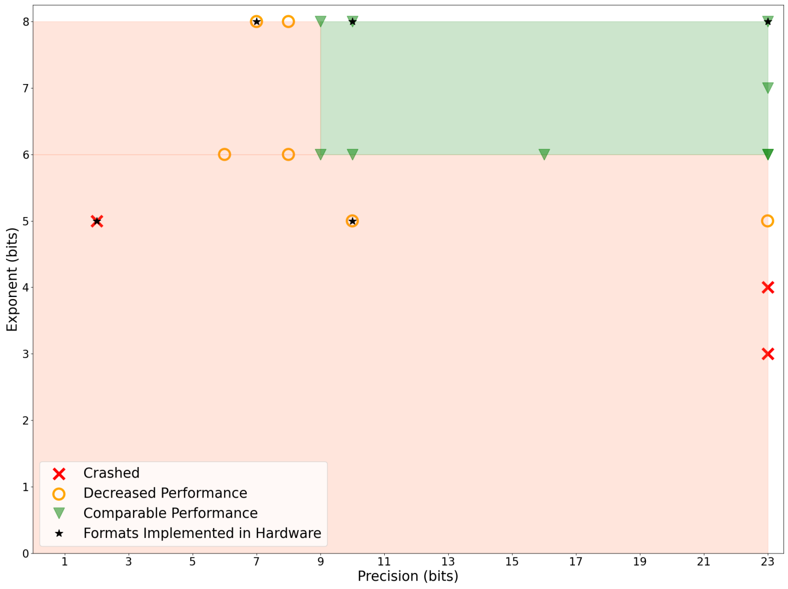

2. The study focuses solely on the inference stage of DeepGOPlus, which is justified based on its importance and likelihood of exposure to noise. However, it would have been beneficial to explore the impact of reduced-precision formats on the model's training phase as well. Training is a critical stage where accuracy and stability are equally important, and understanding the effects of precision reduction during training would provide a more comprehensive analysis.

Answer

Examining the training phase of a CNN poses technical challenges due to the long runtime for training which has an additional overhead due to the instrumentation with MCA. As MCA cannot be applied on a GPU and is not multi-threaded, the training must be run on CPU on a single thread. Plans to reduce this bottleneck involve distributing the training of the model across multiple CPUs using data parallelism.

Extract from Conclusion, line 579, addition to line 581:
Future work could study the numerical stability and potential use of reduced precision in the training step of the DeepGOPlus model as this step is an important determinant of DeepGOPlus' performance.
However, training the DeepGOPlus model with MCA is especially compute intensive. Additional overhead can be expected due to the MCA instrumentation as MCA does not currently support multi-threading and can only function on CPU. Addressing the computational intensity of MCA-instrumented training could involve strategies such as implementing distributed training through data parallelism.

3. The study employs Monte Carlo Arithmetic and VPREC to analyze the impact of reduced-precision formats. While these are valid techniques, it would have been valuable to compare their results against alternative precision reduction approaches, such as quantization or low-bit representations. Such comparisons would help establish the relative effectiveness and limitations of different precision reduction methods.

Answer

VPREC was chosen due to its ability to instrument the model without requiring modification of the source code. This explanation has been added to the section about VPREC along with a citation to a paper that does a comparison of precision reduction approaches.

Modified Use of VPREC Backend for Reduced Precision Emulation section from Materials & Methods, addition to line 260:

Table~\ref{table:1} displays the exact precision and exponent range used to simulate each format in VPREC. In addition to these formats, precision values between the range of 52 and 2 and exponent values between the range of 5 and 11 are simulated using VPREC. Despite lacking hardware implementation, this sweep is done to determine the specific point to which DeepGOPlus inference can be reduced without its performance dropping.

4. The paper briefly mentions numerical stability challenges in DNNs and their sensitivity to noise injection, but it does not elaborate on the specific challenges encountered during the investigation of DeepGOPlus. Providing more details and discussing the implications of these challenges on the model's performance and reliability would enhance the study's contribution.

Answer

Investigating the numerical stability of DeepGOPlus and identifying the challenges it creates is a primary goal of this paper. Since the inference stage was found to be stable, there is not much in terms of challenges to discuss. Therefore in Discussion, section DeepGOPlus Numerical Stability, starting from the third paragraph, the discussion is pivoted to focus on what is contributing to the model's numerical stability. Nonetheless, besides investigating in more detail where performance drops, an additional experiment is being conducted to determine the part of the code that is the source of the limited numerical instability in order to understand potential numerical challenges.

Excerpt from DeepGOPlus Numerical Stability in Discussion, line 463

Therefore, previous concerns about the numerical stability of CNNs as well as other DNNs from the literature ~\cite{kloberdanz2022deepstability, faraone2019monte, haber2017stable, li2020implicit} do not manifest in DeepGOPlus inference, which may be due to several reasons.

First, the relatively shallow architecture of the DeepGOPlus CNN model may favor numerical stability…

5. The study concludes that DeepGOPlus cannot currently be implemented with a lower-precision floating-point format. However, the paper does not provide quantitative performance metrics or an extensive evaluation of the model's accuracy, computational efficiency, and memory consumption under different precision formats. Including such metrics and a comprehensive evaluation would strengthen the conclusions and support the study's claims effectively.

Answer

VPREC is a tool that emulates reduced numerical formats, therefore it can simulate a program's output at reduced precision, but it cannot simulate how a program's performance is impacted. This is why no performance metrics were included in the paper, however an explanation for this has been added to the beginning of the discussion on the Use of VPREC Backend for Reduced Precision Emulation within Materials and Methods.

Modified Use of VPREC Backend for Reduced Precision Emulation section from Materials & Methods, addition to line 260:

Table~\ref{table:1} displays the exact precision and exponent range used to simulate each format in VPREC.

… (Another revision is added before this section) ..

VPREC was chosen due to the ease with which it could be applied and because it does not require any modification of the source code unlike other reduced precision techniques.

VPREC is utilised as an exploratory technique to investigate the possibility of implementing reduced formats. By emulating reduced formats, VPREC allows for us to simulate the

model’s predictions for each format, but it does not simulate the overall performance of the model in terms of computational efficiency or memory consumption.

Such metrics are not analysed in our study as they would not properly reflect the model’s performance at actual reduced precision. In order to physically re-implement DeepGOPlus at reduced precision, other methods should be explored~\cite{cherubin2020tools}, although most DNNs have required source code modifications or recompilation to adjust their precision. Nonetheless, existing work on reduced precision has demonstrated that reducing single-precision to float16 using mixed precision achieved a speedup of 2-6x, while maintaining equivalent performance across a variety of DNNs~\cite{micikevicius2017mixed}.

6. The study focuses on DeepGOPlus, and its findings may not directly generalize to other CNN architectures. It would be valuable to discuss the implications of the study's results and insights in a broader context, addressing how the observed numerical stability and precision requirements may vary for different CNN models used in diverse domains.

Answer

Discussion on how the results of this study can generalise to other CNN structures has been added to the Conclusion section.

Modified Conclusion, addition at line 578:

…In conclusion, while the DeepGOPlus inference stage is stable in its current state, most arithmetic operations cannot have their precision reduced without suffering a substantive drop in performance.

Generalizing these findings to other CNNs presents challenges due to the diversity of possible implementations that can impact numerical stability. Common architectural features like pooling operations may help reduce numerical uncertainty, but software tools and input data types can significantly affect a model's numerical stability. Therefore, further experimental investigations into the stability of different CNNs~\cite{pepe2023numerical}, as well as theoretical explorations of CNN architecture stability are needed to establish more robust generalisations about CNNs.

7. Deep learning is well-known and has been used in previous bioinformatics studies i.e., PMID: 36166351, PMID: 34730875. Therefore, the authors are suggested to refer to more works in this description to attract a broader readership.

Answer

PMID: 36166351, PMID: 34730875, other applications of CNNs in proteomics and models compared to DeepGOPlus in the original paper were cited in addition to the original cited proteomics reviews.

8. The paper provides a link to the project's code repository, which is commendable.

However, it would be beneficial to have a web server to represent their model. This would enhance the reproducibility and enable interested researchers to build upon the work.

Answer

This paper uses the pretrained model provided by the authors of the DeepGOPlus paper and perturbs it with Monte Carlo Arithmetic. This procedure does not result in a new model, only perturbed results, which are in the process of being made available on Zenodo. Nonetheless, the images used to execute the experiments are available on Docker Hub, with instructions on how to find the image on this study's GitHub README.

9. The authors should compare their performance results to previously published works on the same problem/data.

Answer

The Sanity Check section within Results demonstrates that the model used in this study has equivalent performance to the model used by the DeepGOPlus authors, as per the performance metrics they have made available. The results shown are computed on a dataset which contains more recent protein function annotations than those published in the paper. A comparison of performance results to previously published works is available in the DeepGOPlus paper, but these results (including the DeepGOPlus model) were obtained using the less recent dataset and are therefore not comparable.
However, a new row, as seen in the New Table, has been added to demonstrate the difference in metrics when the Diamond tool is included in the prediction scores (DeepGOPlus, IEEE, Verificarlo Python, Verrou TF, Verrou All) vs when it is not (DeepGOPlus CNN).

OLD TABLE – missing comparison to CNN only

Table 3. Classification Metrics from original paper (DeepGOPlus), non-perturbed environment (IEEE), MCA-instrumented environments (Verificarlo Python, Verrou TF, Verrou All). Average values across 10 MCA repetitions for GO Classes MF, CC and BP.

	Fmax			Smin			AUPR		
	**MF**	**CC**	**BP**	**MF**	**CC**	**BP**	**MF**	**CC**	**BP**
DeepGOPlus	0.664	0.695	0.530	9.591	10.442	44.370	0.671	0.738	0.496
IEEE	0.6639578	0.6944381	0.5301602	9.5972334	10.4480327	44.3779111	0.6707084	0.7379463	0.4958606
Verificarlo Python	0.6639578	0.6944381	0.5301602	9.5972334	10.4480327	44.3779111	0.6707084	0.7379463	0.4958606
Verrou TF	0.6639578	0.6944381	0.5301602	9.5972334	10.4480327	44.3779111	0.6707084	0.7379463	0.4958606
Verrou All	0.6639578	0.6944381	0.5301602	9.5972334	10.4480327	44.3779111	0.6707084	0.7379463	0.4958607

NEW TABLE – contains metrics from CNN only

Table 3. Classification Metrics from DeepGOPlus authors (DeepGOPlus), non-perturbed environment (IEEE), MCA-instrumented environments (Verificarlo Python, Verrou TF, Verrou All) and just the CNN itself (DeepGOPlus CNN). Average values across 10 MCA repetitions for GO Classes MF, CC and BP.

	Fmax			Smin			AUPR		
	**MF**	**CC**	**BP**	**MF**	**CC**	**BP**	**MF**	**CC**	**BP**
DeepGOPlus	0.664	0.695	0.530	9.591	10.442	44.370	0.671	0.738	0.496
IEEE	0.6639578	0.6944381	0.5301602	9.5972334	10.4480327	44.3779111	0.6707084	0.7379463	0.4958606
Verificarlo Python	0.6639578	0.6944381	0.5301602	9.5972334	10.4480327	44.3779111	0.6707084	0.7379463	0.4958606
Verrou TF	0.6639578	0.6944381	0.5301602	9.5972334	10.4480327	44.3779111	0.6707084	0.7379463	0.4958606
Verrou All	0.6639578	0.6944381	0.5301602	9.5972334	10.4480327	44.3779111	0.6707084	0.7379463	0.4958607
DeepGOPlus CNN	0.4743216	0.6580121	0.4083806	13.164216	11.807528	49.518513	0.4356199	0.6847218	0.3578813
