# Supplementary material for: Numerical stability of DeepGOPlus inference
Source: PLoS One. 2024 Jan 29;19(1):e0296725. doi: 10.1371/journal.pone.0296725 (PMC10824456; doi:10.1371/journal.pone.0296725)
Supplement: Attachment — Submitted filename: Response_to_Reviewers.pdf [file pone.0296725.s005.pdf]

UNIVERSITÉ Concordia UNIVERSITY

GINA CODY SCHOOL OF ENGINEERING
AND COMPUTER SCIENCE

Department of Computer Science and Software Engineering

Montreal,
November 30, 2023

Dear Editor and Reviewers,

We would like to thank you again for considering our manuscript for publication in *PLOS One* and for the thorough review. Please find below the answer to your comments regarding the second round of comments. The corresponding revision of our manuscript is attached, where revisions are highlighted in blue. All references to line numbers in the response below, correspond to the original unmodified manuscript.

We thank you again for your time and consideration, and we remain available for any further information you may need.

Yours sincerely,

Inés Gonzalez Pepe, on behalf of the authors

Signature

Inés Gonzalez Pepe

Comments to the Author

1. If the authors have adequately addressed your comments raised in a previous round of review and you feel that this manuscript is now acceptable for publication, you may indicate that here to bypass the “Comments to the Author” section, enter your conflict of interest statement in the “Confidential to Editor” section, and submit your "Accept" recommendation.

Reviewer #3: All comments have been addressed

Reviewer #4: (No Response)

Reviewer #5: All comments have been addressed

Reviewer #6: (No Response)

Reviewer #7: All comments have been addressed

---

2. Is the manuscript technically sound, and do the data support the conclusions?

The manuscript must describe a technically sound piece of scientific research with data that supports the conclusions. Experiments must have been conducted rigorously, with appropriate controls, replication, and sample sizes. The conclusions must be drawn appropriately based on the data presented.

Reviewer #3: Yes

Reviewer #4: Yes

Reviewer #5: Yes

Reviewer #6: Yes

Reviewer #7: No

---

3. Has the statistical analysis been performed appropriately and rigorously?

Reviewer #3: I Don't Know

Reviewer #4: Yes

Reviewer #5: Yes

Reviewer #6: Yes

Reviewer #7: No

---

4. Have the authors made all data underlying the findings in their manuscript fully available?

The PLOS Data policy requires authors to make all data underlying the findings described in their manuscript fully available without restriction, with rare exception (please refer to the

Data Availability Statement in the manuscript PDF file). The data should be provided as part of the manuscript or its supporting information, or deposited to a public repository. For example, in addition to summary statistics, the data points behind means, medians and variance measures should be available. If there are restrictions on publicly sharing data—e.g. participant privacy or use of data from a third party—those must be specified.

Reviewer #3: Yes

Reviewer #4: Yes

Reviewer #5: Yes

Reviewer #6: Yes

Reviewer #7: No

---

5. Is the manuscript presented in an intelligible fashion and written in standard English?

PLOS ONE does not copyedit accepted manuscripts, so the language in submitted articles must be clear, correct, and unambiguous. Any typographical or grammatical errors should be corrected at revision, so please note any specific errors here.

Reviewer #3: Yes

Reviewer #4: Yes

Reviewer #5: Yes

Reviewer #6: Yes

Reviewer #7: Yes

---

6. Review Comments to the Author

Please use the space provided to explain your answers to the questions above. You may also include additional comments for the author, including concerns about dual publication, research ethics, or publication ethics. (Please upload your review as an attachment if it exceeds 20,000 characters)

Reviewer #3: My previous comments have been addressed. The manuscript has been improved and can meet the requirement for publication.

Reviewer #4: Numerical Stability of DeepGOPlus Inference

My comments and suggestions are the following:

1.What are the three main advantages and drawbacks of DeepGOPlus as compared to the existing methods?

In consideration of the reviewer’s feedback, the justification for the choice of studying DeepGOPlus in the Introduction at line 36 has been expanded on, as seen below.

This paper focuses its investigation on DeepGOPlus [19], a cutting-edge CNN model for protein function classification. DeepGOPlus, as a computational approach, offers significant advantages such as the rapid processing of extensive protein sequences compared to traditional experimental methods. Notably, it simplifies user interaction by solely requiring the protein sequence as input, eliminating the need for complex additional information about the protein.

While CNN architecture stands as one of the most widely adopted methods for protein function prediction, it is worth acknowledging the success of alternative approaches, including K-Nearest-Neighbors and Logistic Regression~\cite{seo2018deepfam,you2018deeptext2go,you2018golabeler,you2019netgo,wang2023netgo}. Many models in this domain typically incorporate supplementary protein information, such as protein-protein interactions and structural data, to enhance their performance. However, DeepGOPlus was specifically chosen due to being a top performer in the CAFA3 challenge [20], a community-wide assessment of computational protein function prediction methods. Furthermore, the availability of both the DeepGOPlus model and its associated data makes it an ideal choice for this study, facilitating accessibility and further analysis.

2.What are the key benefits of “DeepGOPlus CNN” model as compared to “HGANet” and “CSLNet” models which were presented in these research work (“Automated identification of human gastrointestinal tract abnormalities based on deep convolutional neural network with endoscopic images”) and (“Automated multi-class classification of skin lesions through deep convolutional neural network with dermoscopic images”). Explain briefly main benefits of DeepGOPlus CNN over HGANet and CSLNet in Introduction section.

While HGANet, CSLNet, and DeepGOPlus are all CNN models proposed for various tasks in the medical field, it's important to note that they are specifically designed for different tasks than protein prediction and employ distinct architectural approaches.

Our study focuses on protein function prediction, therefore we believe it is essential to maintain a coherent and unambiguous narrative within our field of interest. This will ensure that our readers are not confused by the inclusion of unrelated models.

In consideration of the feedback, however, the Conclusion was modified as of line 630:

Furthermore, studying deeper CNNs than DeepGOPlus, where the risk of exploding or vanishing gradients [54] is prevalent and may affect numerical stability, could yield insights into the numerical properties of CNNs in a broader context. Owing to the widespread adoption of CNNs in various domains, the practice of employing MCA during CNN inference to investigate numerical stability has extended beyond its original application to DeepGOPlus and can now be used in other fields, including medical imaging~\cite{pepe2023numerical}.

3. It is suggested to authors to make a detailed block diagram or flowchart of proposed method so that the readers can easily understand it.

In light of the proposed feedback, Figure 1 in the paper, which is meant to illustrate the proposed method, has been modified in order to better clarify the method for the readers.

OLD

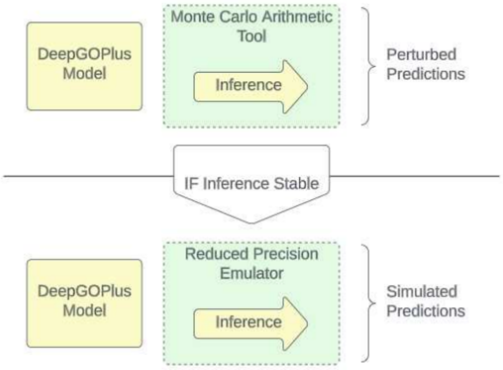

Figure 1: Methods summary

NEW

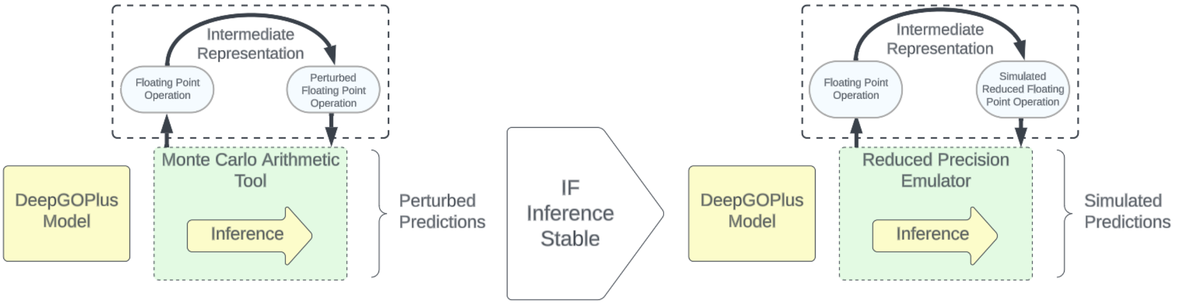

4. It is suggested to the authors to use an official email address instead of “@gmail.com”.

Changed to i_gon@encs.concordia.ca

5.To establish the numerical stability of DeepGOPlus, authors quantified the numerical uncertainty of floating point model using Monte Carlo Arithmetic. Are there other techniques instead of Monte Carlo?

In light of the reviewer’s feedback, we propose the following changes to the Introduction as of line 47.

Other approaches to determining numerical stability involve formal error analysis and interval arithmetic [22], but unlike MCA they do not scale to large code bases and require modification of the source code. Moreover, MCA is chosen over another stochastic arithmetic technique, Discrete Stochastic Arithmetic (DSA)~\cite{vignes2004discrete}, as DSA is less scalable to large code bases and makes limitative assumptions about the nature of the distribution of computational errors.

Reviewer #5: In my estimation, you have largely succeeded in improving the manuscript in accordance with the reviewers' comments.

The article now reads more comprehensive and well-rounded overall.

In light of its strengths, primarily relevance, novelty and thoroughness in terms of its stated objective, I am hereby recommending approval for publication.
NOW THE PAPER IS SUITABLE FOR PUBLICATION

Reviewer #6: The article delves into a fundamental aspect for the generation of robust and reliable deep learning models: the evaluation of numerical stability. As the authors rightly point out, this is particularly important in the case of determining a protein's function via deep learning. This evaluation assesses how resilient a model is when subjected to minor numerical perturbations during calculations. The article is well-researched, with a detailed analysis and the application of various verification methods. In terms of its structure, it is comprehensive, clear, and instructive. Overall, it is a well-executed article with intriguing and thought-provoking results. I want to emphasize the diligent work of the authors.
I would like to offer a few minor suggestions to enhance the understanding of certain aspects of the article and provide some observations regarding minor structural aspects of the article.

-In my personal opinion, I would remove the quote "The code for this project can be found at https://github.com/big-data-lab-team/deepgoplus-stability" from the abstract and place it in a specific section, but not in the abstract.
The line about the code repository had been moved to the Dataset section, now renamed Data and Code Availability.

-Probably, I would consolidate all the links to data and code used in the article in a separate section, regardless of whether access is referenced in different sections.
The links have been consolidated to the newly renamed Data and Code Availability section (formerly Dataset).

-Around line 30 ("This paper centers its investigation on DeepGOPlus [19], a state-of-the-art CNN protein function classification model. It is chosen due to being one of the top performers in the CAFA3 challenge [20], a community-wide challenge to assess computational protein function prediction methods."), it might be desirable to delve further into the selection of DeepGoPlus as the model of choice. An explanation in this regard or a reference to more recent developments could be interesting, including a more quantitatively substantiated justification. Please consider adding one or more paragraphs where you compare it to these alternatives and the added value of your choice.
In consideration of this feedback, an explanation about the motivation behind the selection of DeepGOPlus has been included in the introduction as shown <u>here</u> in our response to Reviewer 4's comments.

-It would be interesting to include in DeepGOPlus Model Section an image that provides a schematic of the architecture of the DNN model used. Perhaps, placing it around line 286 would be beneficial. This would assist the reader in understanding the network's structure.
As recommended, a diagram of the DeepGOPlus model's architecture, from the original paper, has been included in the DeepGOPlus Model section.

-A "Future Work" section that compiles the future phases of this research would be beneficial. For instance, the content from line 620 onwards is likely better placed in a dedicated section rather than within the "Conclusions".
As recommended, the content from line 620 has become a new section titled Future Work.

Reviewer #7:

1. The authors must provide statistical values related to DeepGOPlus inference time before and after reduced precision floating point formats, memory consumption, and latency values. Provide all the details in a Table format.

In consideration of the reviewer's feedback, tables have been added to the paper in the Appendix B, detailing the runtime and maximum resident set size as a measure of memory consumption observed across the perturbed and non-perturbed environments. We refer to it at line 344 within the Sanity Check section.

As an additional sanity check, we measure the runtimes and memory consumption of our method, with and without instrumentation to ensure we are not creating an excessive overhead in terms of computational resources. In Appendix~\ref{appendix_b}, we display these performance metrics and confirm instrumentation with MCA simply adds a slowdown factor to the project.

2. How the current work is novel and highlight the contributions.

In order to clarify this study's contributions, we propose the following modifications to the Introduction, as of line 56.

We evaluate reduced precision formats that have existing hardware implementations to increase the practical value of our results. Our contributions can be summarised as follows:

1) Quantification of the numerical stability in the DeepGOPlus CNN's inference stage, providing a reliability metric for model users.
2) Exploration of the impact of reduced precision on the performance of the DeepGOPlus CNN through simulation.

3. There are several LSTM techniques for sequence-based predictions; authors must justify the reason for considering CNN suitable for classification tasks.

In consideration of this feedback, an explanation about the motivation behind the selection of DeepGOPlus has been included in the introduction as shown <u>here</u> in our response to Reviewer 4's comments.

4. Authors must mention the computational complexity of the model related to state-of-the-art techniques with hardware specifications such as GPU RAM system configurations.

In response to the author's suggestions, we modified the Reduced Precision for DNNs section at line 113 to include a discussion on CNN computational complexity.

DNNs are becoming widely adopted, but one of their limitations is the amount of computing resources required for their training and deployment.

The computational complexity of a CNN is often determined by the number of arithmetic operations it does. This is mathematically represented by~\cite{nakata2021adaptive} as:

$$\#\ of\ arithmetic\ operations\ = O_h O_w C_o C_i K_h K_W$$

Where O_h and O_w refer to the height and width of the output feature maps, C_o and C_i are the number of output and input channels, and K_h and K_w are the height and width of kernels.

However, this is just a general equation as the number of arithmetic operations can be

impacted by pooling layers used in a CNN as well as the algorithmic approach to doing certain arithmetic operations. Nonetheless, while reduced precision does not cut down on the computational complexity of the CNN, it has been shown to decrease the resource consumption of the model, which is why it is of interest in this study.

5. Authors have just combined a few existing techniques. Is it sufficient for a standard journal? Please justify the same.
Not about approach but about application + understanding deepgoplus
Better understand numerical properties about other real world applications – mention in intro

The objective of this study is not to introduce a novel technique but rather to leverage an existing approach, specifically Monte Carlo Arithmetic, in a practical real-world context to analyse numerical uncertainty. Historically, the study of numerical uncertainty has been confined to limited, small-scale scenarios due to the constraints of available methodologies. In this research, we adopted a state-of-the-art model with broad potential utility for numerous researchers and assessed the applicability of MCA in more comprehensive real-world scenarios, surpassing prior examinations. Our achievement lies in not only quantifying the numerical uncertainty of the DeepGOPlus CNN to provide a measure of reliability but also in exploring the potential of harnessing the model's stability through simulated reduced precision assessments.

Furthermore, we modified the Introduction, starting at line 20, to better clarify the need to understand the numerical properties of real world applications.

Nonetheless, while current DNN models are able to achieve high predictive performance, the numerical reliability of these predictions — defined as the stability with respect to minor numerical perturbations — is unknown and should be evaluated for several reasons. Considering DNNs often operate as “black boxes” for which we lack a full understanding, it is essential to understand their behaviour or at least their numerical properties. With this objective in mind, our aim is to delve into the numerical stability of a DNN, not only to gauge its reliability but also to shed light on its underlying numerical properties.
