# Supplementary material for: Numerical stability of DeepGOPlus inference
Source: PLoS One. 2024 Jan 29;19(1):e0296725. doi: 10.1371/journal.pone.0296725 (PMC10824456; doi:10.1371/journal.pone.0296725)
Supplement: Attachment — Submitted filename: comments_PONE-D-23-12824.docx [file pone.0296725.s004.docx]

**Numerical Stability of DeepGOPlus Inference**

My comments and suggestions are the following:

1. What are the three main advantages and drawbacks of DeepGOPlus as compared to the existing methods?
2. What are the key benefits of “DeepGOPlus CNN” model as compared to “HGANet” and “CSLNet” models which were presented in these research work (“Automated identification of human gastrointestinal tract abnormalities based on deep convolutional neural network with endoscopic images”) and (“Automated multi-class classification of skin lesions through deep convolutional neural network with dermoscopic images”). Explain briefly main benefits of DeepGOPlus CNN over HGANet and CSLNet in Introduction section.
3. It is suggested to authors to make a detailed block diagram or flowchart of proposed method so that the readers can easily understand it.
4. It is suggested to the authors to use officials email address instead of “@gmail.com”.
5. To establish the numerical stability of DeepGOPlus, authors quantified the numerical uncertainty of floating point model using Monte Carlo Arithmetic. Are there other techniques instead of Monte Carlo?
